# An Account of Diseases in an Eastern District of London, from the 20th of August, to the 20th of September, 1811

**Published:** 1811-10

**Authors:** 


### *An Account of Diseases in an Eastern District of London, from the 20th of August, to the 20th of September, 1811.*

| | |
|---|---|
| **ACUTE DISEASES.** | |
| Typhus | 5 |
| Scarlatina | 3 |
| Peripneumonia notha | 2 |
| Rheumatismus acutus | 2 |
| **CHRONIC DISEASES.** | |
| Tussis | 7 |
| Dyspnœa | 4 |
| Tussis cum Dyspnœa | 6 |
| Pleurodyne | 3 |
| Phthisis Pulmonalis | 2 |
| Hydrothorax | 2 |
| Ascites | 2 |
| Amenorrhœa | 4 |
| Leucorrhœa | 5 |
| Dysuria | 4 |
| Chlorosis | 3 |
| Hepatitis Chronica | 1 |
| Rheumatismus Chronicus | 5 |
| **PUERPERAL DISEASES.** | |
| Menorrhagia Lochialis | 4 |
| Dolor post partum | 7 |
| Dysuria | 3 |
| **INFANTILE DISEASES.** | |
| Convulsio | 2 |
| Herpes | 5 |
| Tinea | 3 |
| Dentitio | 2 |

The disease which was particularly referred to in the last Report has for some time appeared less frequently. Scarlatina, though it still still occasionally appears, seems to have lost the character of an epidemic. It cannot have escaped the observation of the attentive practitioner, that this disease is frequently succeeded by dropsical symptoms; and this has been exemplified in some of the instances referred to. In these, however, they have been mild, and no circumstance has yet occurred to produce any alarm respecting the termination of the complaint. There have been some appearances of a less common kind in some other cases; an eruption has taken place in different parts of the body, which has in two or three instances been very unpleasant in its appearance, and very difficult to cure. On the lips and nose of one child it spread so far as to change the character of the countenance, and materially disfigure him; and in another instance the right arm and hand were covered by a number of small ulcers, which were attended by an intolerable itching. These symptoms are likely gradually to subside, and may have proved a critical termination of the original disease, and have prevented what might have proved of more serious consequence to the health of the patients, or might even have endangered the life.

